# Analysis of oxidized glucosylceramide and its effects on altering gene expressions of inflammation induced by LPS in intestinal tract cell models

**DOI:** 10.1038/s41598-023-49521-3

**Published:** 2023-12-18

**Authors:** Mirinthorn Jutanom, Shunji Kato, Shinji Yamashita, Masako Toda, Mikio Kinoshita, Kiyotaka Nakagawa

**Affiliations:** 1 Food Function Analysis Laboratory, Graduate School of Agricultural Science, Tohoku University, 468-1 Aramaki Aza Aoba, Aoba-ku, Sendai, Miyagi 980-8572 Japan; 2 Department of Molecular Pathobiology, Faculty of Pharmaceutical Sciences, Kyushu University, 3-1-1 Maidashi Higashi-ku, Fukuoka, 812-8582 Japan; 3 Department of Life and Food Sciences, Obihiro University of Agriculture and Veterinary Medicine, Obihiro, 080-8555 Japan; 4 Food and Biomolecular Science Laboratory, Graduate School of Agricultural Science, Tohoku University, Sendai, Miyagi 980-8572 Japan

**Keywords:** Sphingolipids, Lipid peroxides, Inflammatory bowel disease

## Abstract

Glucosylceramide (GlcCer) belongs to sphingolipids and is found naturally in plant foods and other sources that humans consume daily. Our previous studies demonstrated that GlcCer prevents inflammatory bowel disease both in vitro and in vivo, whose patients are increasing alarmingly. Although some lipids are vulnerable to oxidation which changes their structure and activities, it is unknown whether oxidative modification of GlcCer affects its activity. In this research, we oxidized GlcCer in the presence of a photosensitizer, analyzed the oxide by mass spectrometric techniques, and examined its anti-inflammatory activity in lipopolysaccharide (LPS)-treated differentiated Caco-2 cells as in vitro model of intestinal inflammation. The results showed that GlcCer is indeed oxidized, producing GlcCer hydroperoxide (GlcCerOOH) as a primary oxidation product. We also found that oxidized GlcCer preserves beneficial functions of GlcCer, suppressing inflammatory-related gene expressions. These findings suggested that GlcCerOOH may perform as an LPS recognition antagonist to discourage inflammation rather than induce inflammation.

## Introduction

Glucosylceramide (GlcCer) is composed of a long-chain base (LCB) with an amide-link fatty acid (i.e., ceramide (Cer)) and a polar head group (i.e., glucose (Glc)), one class of sphingolipids and is found naturally in plant-derived foods. The LCB composition of GlcCer contained in edible plants is diverse and depends on plant species; for instance, the predominant bases of GlcCer are *trans*-4, *cis*-8-sphingadienine (d18:2^4*E*,8*Z*^) in rice and maize, d18:2^4*E*,8*E*^ in soybeans, and d18:1^8*Z*^ in wheat and rye^1^. Several cell culture and animal studies have reported that GlcCer has a certain ability to ameliorate abnormal lipid metabolism and atopic dermatitis^2–4^. Since we consume a certain amount of GlcCer from plant foods such as rice, maize, and other sources, for example, a daily Japanese diet contains approximately 50 mg of GlcCer^5,6^, it is believed that GlcCer is consumed in the diet can help prevent these diseases^7^.

Besides the above, to these beneficial functions of GlcCer, our research group speculated if GlcCer might also have additional functions and thus conducted cell culture experiments to explore them. As a model of intestinal inflammation, we treated differentiated human intestinal Caco-2 cells with lipopolysaccharide (LPS) and then with plant GlcCer prepared from maize and found that GlcCer significantly inhibited LPS-induced inflammation^8^. Together with the results of animal studies^9,10^, it is suggested that GlcCer ingestion prevents LPS-induced inflammation in the intestine, thereby preventing inflammatory bowel disease (IBD) and the formation of abnormal crypt foci in the colon, a cause of colon cancer, which is a worldwide problem, potentially.

It is well known that lipids are susceptible to oxidization due to their double bond, which might alter their functions. Thus, like other lipids^11^, GlcCer might undergo oxidation and alter its activities by modifying its structure. Previous studies have reported that GlcCer-related compounds (i.e., galactosylceramide (GalCer) and lactosylceramide (LacCer)) undergo structural modifications due to oxidation^12,13^. For example, Santinha et al.^12^ reported that photooxidation of GalCer and LacCer can produce some oxides with hydroperoxyl group. Furthermore, Couto et al.^13^ showed that radical oxidation of GalCer and LacCer forms oxides with groups such as keto, hydroxy, and hydroperoxyl. We hypothesized that GlcCer, as well as GalCer and LacCer, could undergo oxidative modification to become oxidized GlcCer and thereby lose GlcCer activity (e.g., alleviating inflammatory effects). Alternatively, the exact opposite possibility might be considered. Since certain oxidants are known to have beneficial effects^14–16^ (e.g., oxidized phospholipids have been reported as antagonists of LPS rather than inducers of inflammation^17–19^), oxidation of GlcCer may promote its activity. To the best of our knowledge, no research from such a perspective has been done in the past.

To prove the above hypothesis, in this study, we first attempted to oxidize GlcCer by photooxidation in the presence of a photosensitizer. We then analyzed the oxide by quadrupole time-of-flight mass spectrometry (qTOF-MS) and liquid chromatography-tandem mass spectrometry (LC–MS/MS). The primary oxidation product of GlcCer was identified and collected. Their anti-inflammatory activity in LPS-treated differentiated Caco-2 cells was examined compared to the original GlcCer to determine whether the structure of GlcCer affects its action. This research demonstrated that GlcCer is indeed oxidized, but the modification does not diminish its anti-inflammatory properties. These findings may provide useful information for further utilization of GlcCer as a functional food element in the future.

## Experimental section

### Chemicals and materials

Rice-derived GlcCer, ≥ 99%(TLC) was obtained from Nippon Flour Mills Co., Ltd., (Atsugi, Japan). Rose bengal (RB) was purchased from Fujifilm Wako Pure Chemical Co. (Osaka, Japan). Human intestinal epithelial (Caco-2) cell models derived from colon carcinoma were purchased from ATCC (Manassas, USA). LPS from *E. coli* 055, Dulbecco’s modified Eagle medium (DMEM), non-essential amino acid solution (NEAA), penicillin–streptomycin solution (P/S), trypsin, and fatty acid-free bovine serum albumin (BSA) were obtained from Fujifilm Wako Pure Chemical Co. Fetal bovine serum (FBS) was purchased from Gibco (Tokyo, Japan). All other reagents were of the highest grade available.

### GlcCer oxidation and qTOF-MS analysis

GlcCer (25 mg) was dissolved in 5 mL ethanol and placed in a glass vial tube to which a photosensitizer (0.05 mg RB) was added. After the glass vial tube was completely closed, the sample solution was incubated at 4 °C for up to 120 h under an 18 W LED light of 50 Klux (10 cm vertically above the glass vial tube). The resulting solution was loaded onto a SepPak Plus QMA cartridge (Waters, Milford, USA), equilibrated with ethanol, unoxidized and oxidized GlcCer were eluted with 5 mL of ethanol; RB was retained on the cartridge. The eluate was evaporated and dissolved in 5 mL methanol. A portion of the solution (10 μL) was then dissolved in 1 mL methanol and infused directly into qTOF-MS (Bruker Daltonics GmbH, Bremen, Germany) at a flow rate of 10 μL/min (Table S1) to ensure the formation of oxidized GlcCer.

### LC–MS/MS analysis of oxidized GlcCer samples

One μL from the above 5 mL methanol sample was diluted with 1 mL methanol, and 20 μL of it was analyzed by LC–MS/MS (4000QTRAP (SCIEX, Tokyo, Japan)). Product ion scanning was performed at *m/z* 824.8 to assess whether oxidized GlcCer (i.e., GlcCer hydroperoxide (GlcCerOOH)) was present in the sample. An ODS column (COSMOSIL 5C18-MS-II, 2.0 × 250 mm, Nacalai Tesque, Kyoto, Japan) was used with methanol/water (95:5, v/v) containing 0.1% formic acid as the mobile phase at a flow rate of 0.2 mL/min. The column temperature was maintained at 40 °C. Other parameters are shown in Table S2. After confirming the presence of GlcCerOOH, each GlcCerOOH isomer was specifically analyzed by LC–MS/MS using multiple reaction monitoring (MRM). The MRM pair for each GlcCerOOH isomer (Table S3) was determined based on the product ion scan data. Analysis conditions were the same as above.

### Preparation of the mixture of GlcCerOOH isomers

GlcCer (25 mg) was oxidized for 96 h as described above. This sample was subjected to high-performance liquid chromatography with a UV detector (HPLC–UV) (210 nm), and the GlcCerOOH isomer fraction was collected. ODS column (COSMOSIL 5C18-MS-II, 4.6 × 250 mm, Nacalai Tesque) was used with methanol/water (95:5, v/v) as the mobile phase at a flow rate of 1 mL/min. The column temperature was maintained at 40 °C. The composition of the fractionated GlcCerOOH isomer mixture was examined by LC–MS/MS. The solvent of the GlcCerOOH isomer mixture was evaporated and dissolved in an appropriate amount of ethanol. The total amount of GlcCerOOH isomers in ethanol was measured by ferrous oxidation-xylenol orange (FOX) 2 assay^20^. The prepared GlcCerOOH isomer mixture was stored in the dark at – 80 °C until used for cell culture experiments.

### Cell culture

Human intestinal Caco-2 cells derived from colon carcinoma (ATCC, Manassas, USA) were cultured in DMEM supplemented with 10% heat-inactivated FBS (v/v), 1% P/S (v/v), and 1% NEAA (v/v) in an incubator at 37 °C containing 5% CO_2_ under humid conditions. Caco-2 cells (1.5 × 10^6^ cells/mL) were continuously passaged every 3–4 days, and the medium was changed every 2 days. Differentiated Caco-2 cells were obtained by seeding Caco-2 cells; when > 90% confluence was reached, the cells were designated as day 0 and cultured for 21 days to induce differentiation. Then, the cells were treated with 50 μg/mL LPS to induce inflammatory responses.

### Cell viability

Human intestinal Caco-2 cells were cultured in 96-well plates containing 100 μL of culture medium with 10% FBS until differentiation. Then, the medium of differentiated Caco-2 cells (5.0 × 10^4^ cells/mL) was changed to DMEM containing 0.1% BSA with/without LPS (50 μg/mL) and GlcCer or GlcCerOOH isomers (5, 10, 20, 50 μM). After 48 h of incubation, 10 μL of cell counting kit-8 (CCK-8) solution (Dojindo, Kumamoto, Japan) was added and incubated for another 2 h, and absorbance was measured at 450 nm using a microplate reader^21^.

### RT-qPCR

Differentiated Caco-2 cells (2.5 × 10^6^ cells) were treated with GlcCer or GlcCerOOH isomers (20, 50 μM) either with or without LPS (50 μg/mL) for 24 h. Total mRNA was extracted using a NucleoSpin® RNA (Takara Bio Inc., Shiga, Japan). PrimeScript Master Mix (Takara Bio Inc.) was used to synthesize cDNA from total RNA (500 ng) following the manufacturer’s instructions. PCR amplification was performed with a CFX96 Ex Taq II (Takara Bio Inc.) using gene-specific primers (Sigma-Aldrich, Tokyo, Japan). PCR conditions were 95 °C for 30 s, followed by 40 cycles of 95 °C for 5 s and 60 °C for 30 s. The relative expression of each targeted gene was determined using the 2^−ΔΔCT^ method with the housekeeping gene (glyceraldehyde-3-phosphate dehydrogenase (GAPDH)) as the reference gene. The primers used in the real-time quantitative PCR (RT-qPCR) are listed in Table 1.

**Table 1:** Primers sequences of target genes for RT-qPCR.

Gene name	GenBank accession number	Forward primer (5′-3′)	Reverse primer (5′-3′)
Tumor necrosis factor α (*TNF-α*)	NM_000594	TCTCGAACCCCGAGTGACAA	TATCTCTCAGCTCCACGCCA
Nuclear factor kappa β (*NF-κβ*)	NM_003998.4	TGGAGTCTGGGAAGGATTTG	CGAAGCTGGACAAACACAGA
Interleukin 1β (*IL-1β*)	NM_000576.3	CCAGGGACAGGATATGGAGCA	TTCAACACGCAGGACAGGTACAG
Interleukin 4 (*IL-4*)	NM_000589.4	TCATTTTCCCTCGGTTTCAG	AGAACAGAGGGGGAAGCAGT
Interleukin 6 (*IL-6*)	NM_000600.5	ATGAACTCCTTCTCCACAAGCGC	GAAGAGCCCTCAGGCTGGACTG
Prostaglandin-endoperoxide synthase 2 (*COX-2*)	NM_000963.4	TGGCTACAAAAGCTGGGAAG	GCTGCTTTTTACCTTTGACACC
Cyclin-dependent kinase inhibitor 1 (*p21*)	NM_053056.3	CGATGCCAACCTCCTCAACGA	TCGCAGACCTCCAGCATCCA
Tumor protein p53 (*p53*)	NM_000546	GATGCTGTCCGCGGACGATAT	GTGCAAGTCACAGACTTGGC
Glyceraldehyde-3-phosphate dehydrogenase (*Gapdh*)	NM_002046	TCATGGGTGTGAACCATGAGAA	GGCATGGACTGTGGTCATGAG

### Apoptosis detection

Caco-2 cells were cultured in eight-well Lab-Tek chamber slides (Thermo Scientific, Waltham, USA) containing 0.5 mL of culture medium with 10% FBS until differentiation. Then, the differentiated Caco-2 cells (1.0 × 10^6^ cells/mL) medium was changed to DMEM with 0.1% BSA containing 20 μM of GlcCer or GlcCerOOH isomers with LPS (50 μg/mL). After 24 h of incubation, apoptotic cells were visualized under a fluorescence microscope using TACS 2 TdT-Flour in situ apoptosis detection kit (TUNEL assay, Trevigen, Gaithersburg, USA), followed by 4′,6-diamidino-2-phenylindole (DAPI) staining^22,23^. The percentage of apoptotic cells was calculated by dividing the number of apoptotic cells by the total number of cells and multiplying the value by 100. Apoptosis-related genes were evaluated by RT-qPCR as mentioned above.

### Cytokine secretion analysis

The cytokine levels of IL-1β, IL-6, and TNF-α in the cell culture supernatant were quantified using an ELISA Max™ Deluxe Set Human IL-1β (Cat No. 437004), IL-6 (Cat No. 430504), and TNF-α (Cat No. 430204) (Biolegend, Way San Diego, USA). Briefly, differentiated Caco-2 cells (2.5 × 10^6^ cells) were incubated with DMEM containing 0.1% BSA with/without LPS (50 μg/mL) and GlcCer or GlcCerOOH isomers (20, 50 μM) for 24 h. Then, the cell media was collected and added to 96-well microplates with the standards, and the assay was performed according to the manufacturer’s protocol. Absorbance was measured at 450 nm and 570 nm using a 96-well microplate reader (Tecan Group Ltd., Switzerland).

### Statistical analysis

Data are shown as mean ± standard error (SE). All data were subjected to analyses performed with IBM-SPSS statistic version 26 (IBM SPSS Inc, Chicago, USA). Differences between two groups (control vs. LPS group) determined using Student’s *t-*test*.* Differences between among more than three groups were determined using a one-way analysis of variance (ANOVA) with Tukey’s post hoc test. *p*-Value less than 0.05 was considered statistically significant.

## Results and discussion

### qTOF-MS and LC–MS/MS analysis of GlcCer oxidation products

GlcCer, a main sphingolipid class contained in plant-based foods^24^, is shown to impact lipid metabolism positively and exhibits potential benefits in inflammation-related conditions such as inflammatory skin disease^2–4,8^. Previously, oxidation of GlcCer-related compounds (GalCer and LacCer) has been reported^12,13^. Thus, we hypothesize that GlcCer might undergo oxidation, affecting its functions. Regarding rice-derived GlcCer, which accounts for a significant proportion of the GlcCer humans consume daily, the predominant LCB is d18:2^4*E,*8*Z*^. These double bonds would be the target for oxidation. To prove this hypothesis, we subjected rice-derived GlcCer to photooxidation in the presence of photosensitizer to determine whether the ene-reaction of GlcCer with singlet oxygen yields oxides such as hydroperoxide. In general, this ene-reaction has the advantage over radical oxidation in that it does not require higher temperatures and avoids reactions that would decompose the generated oxides.

**Figure 1:**
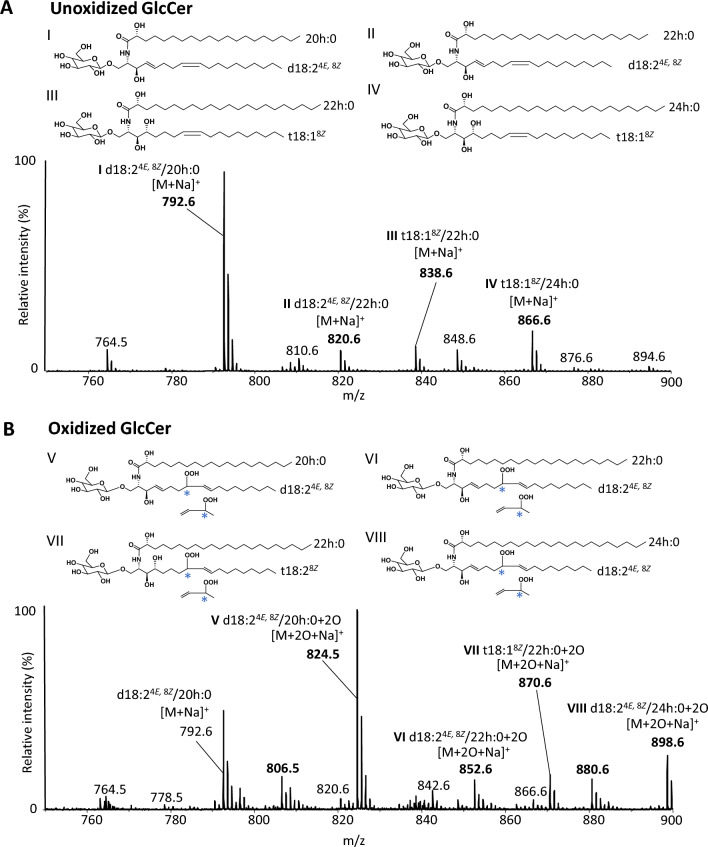
Q1 mass spectra of unoxidized GlcCer (**A**) and oxidized GlcCer (**B**). GlcCer was oxidized in the presence of RB at 4 °C for 96 h under 18 W LED light of 50 Klux. The resulting sample was analyzed by qTOF-MS. Unoxidized GlcCer was also analyzed. Details are shown in the Methods section. *Asterisks* are shown to show the possibility of 8-OOH-GlcCer and 9-OOH-GlcCer, typical GlcCerOOH isomers generated by photo-oxidation.

The samples before and after the photooxidation were measured by qTOF-MS (positive electrospray ionization (ESI)). In the Q1 mass spectrum of unoxidized GlcCer, we observed mainly *m/z* 792.6 [M + Na]^+^, which is considered the molecular species of d18:2^4*E,*8*Z*^/20h:0. The *m/z* 820.6, 838.6, and 866.6 were considered to be molecular species of d18:2^4*E,*8*Z*^/22h:0, t18:1^8*Z*^/22h:0, and t18:1^8*Z*^/24h:0, respectively (Fig. 1A). These observations were in agreement with the previous reports analyzing rice-derived GlcCer^25^. As we expected, the photooxidation of GlcCer yielded new mass shift ions of + 32 Da at *m/z* 824.5, 852.6, 870.6, and 898.6 (Fig. 1B), suggesting the formation of hydroperoxides^12^. However, perhaps these are not hydroperoxides but oxides such as epoxy-hydroxides, dihydroxides, and keto derivatives^13,26,27^ (especially the ions at *m/z* 852.6 and the other *m/z* 880.6 may have keto groups). Therefore, to confirm the formation of GlcCerOOH, we focused on the *m/z* 824.5 ion that was observed abundantly in the Q1 mass spectrum (Fig. 1B) and performed its product ion analysis using LC–MS/MS.

**Figure 2:**
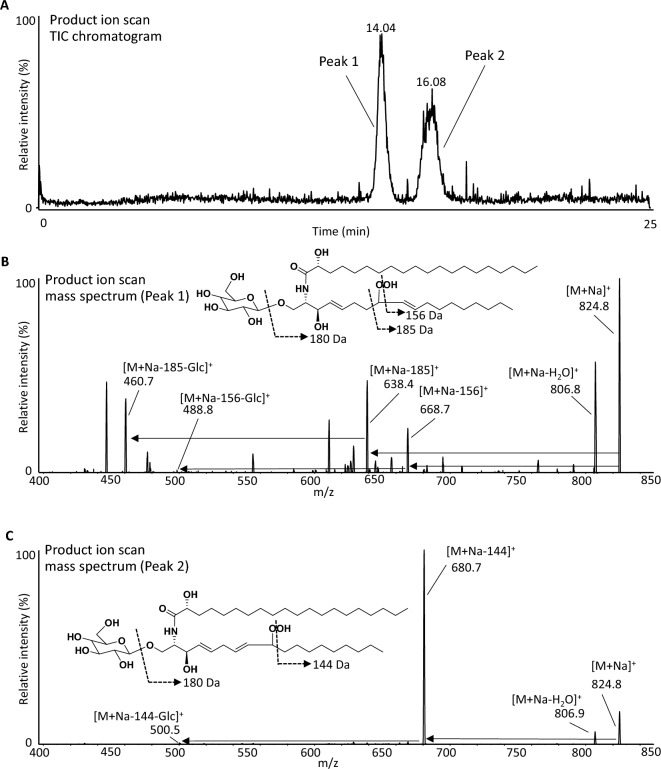
Mass chromatogram and mass spectra obtained from product ion scan. GlcCer was oxidized in the presence of RB at 4 °C for 96 h under 18 W LED light of 50 Klux. The resulting sample was analyzed by LC–MS/MS. Product ion scanning was performed at *m/z* 824.8. Details are shown in the Methods section. Total ion current chromatogram (**A**), mass spectra of peak 1 (13.7–14.5 min) (**B**), and peak 2 (15.5–16.7 min) (**C**).

**Figure 3:**
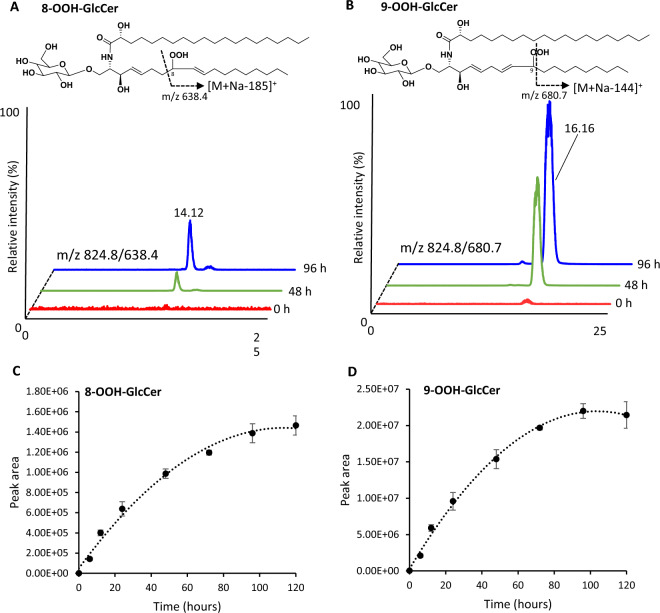
MRM chromatograms (**A**, **B**) and time-dependent changes (**C**, **D**) of GlcCerOOH isomers (8-OOH-GlcCer and 9-OOH-GlcCer). GlcCer was oxidized in the presence of RB at 4 °C for up to 120 h under 18 W LED light of 50 Klux. The resulting sample was analyzed by LC–MS/MS with MRM. Details are shown in the Methods section. Values are mean ± SE, n = 4.

With regard to product ion analysis, our previous LC–MS/MS research demonstrated that collision-induced dissociation (CID) of sodium adduct of lipid hydroperoxide provided the selective fragmentation of hydroperoxyl group using phospholipid^28–30^ and triacylglycerol^31^. Accordingly, the LC–MS/MS method using sodium was considered to be useful in the structural analysis of GlcCerOOH in terms of investigating the presence or absence of hydroperoxyl groups and binding positions with high precision. When the sample of photooxidized GlcCer was analyzed by LC–MS/MS, two peaks were observed at 14.04 and 16.08 min in the product ion chromatogram of *m/z* 824.8 [M + Na]^+^ (Fig. 2A). The mass spectra of the first peak showed that main fragment ions were based on neutral loss (NL) of 156 Da and 185 Da (Fig. 2B). Considering our previous studies, which demonstrated sodium-induced α-cleavage to hydroperoxyl group^32^, the first peak would be GlcCerOOH bearing hydroperoxyl group at the C8 position (i.e., 8-OOH-GlcCer). On the other hand, the second peak at 16.08 min was predominantly composed of NL of 144 Da (Fig. 2C). Therefore, the second peak was considered to be isomer bearing at the C9 position (i.e., 9-OOH-GlcCer) (Fig. 2A), which agreed with the fact that the fragmentation between C9-C10 is more preferred rather than that of C8-C9. In addition, both GlcCerOOH isomers (8-OOH-GlcCer and 9-OOH-GlcCer) showed an NL of the Glc residue (− 180 Da) in the cleavage of a glycosidic bond (*m/z* 460.7 and 488.8 for 8-OOH-GlcCer, and *m/z* 500.5 for 9-OOH-GlcCer) (Fig. 2B, C)^33,34^. By using LC–MS/MS with MRM analysis, we could selectively detect both 8-OOH-GlcCer (*m/z* 824.8/638.4) and 9-OOH-GlcCer (*m/z* 824.8/680.7) (Fig. 3A, B) and found that 8-OOH-GlcCer and 9-OOH-GlcCer were increased depending on oxidation time and reached the plateau point after 96 h (Fig. 3C, D). To our knowledge, this is the first study to show such a formation profile of GlcCerOOH. Finally, a mixture of GlcCerOOH isomers (i.e., 8-OOH-GlcCer and 9-OOH-GlcCer) was collected using HPLC–UV for the subsequent cell culture study (its purity was confirmed by LC–MS/MS (Fig. S1)).

On the other hand, for reference, despite the fact that electrophilic ^1^O_2_ directly reacts with double bond by the ene-reaction and forms hydroperoxides^35^, we did not detect hydroperoxyl derivatives of GlcCer bearing at 4,5-double bond in LCB. To further validate this observation, we analyzed d-erythro-sphingosine (d18:1^4*E*^) photooxidized under the same conditions as GlcCer. As well as GlcCer oxidation, the hydroperoxyl derivatives of D-erythro-sphingosine bearing 4,5-double bond were not observed (Fig. S2). Given these results and the results of the previous study^36^, it is suggested that the presence of the hydroxyl group (C3) in LCB prevents the ^1^O_2_ oxidation of its adjacent 4,5-double bond. This result also appears to be the same for radical oxidation^13,27^.

### Comparison of anti-inflammatory activity of GlcCerOOH with unoxidized GlcCer

LPS is known to modify various signal transductions (e.g., inflammatory cytokine expression) via toll-like receptor 4 (TLR4) in intestinal Caco-2 cells, leading to intestinal inflammation^37,38^. Intestinal inflammation is also known to activate the production of apoptotic proteins and induce apoptosis, which is this phenomenon has been observed in patients with IBD^39,40^. As mentioned in the introduction, in our previous cell culture studies^8,41^, we found that treatment of differentiated Caco-2 cells with unoxidized GlcCer along with LPS significantly suppressed LPS-induced inflammation and even apoptosis. As some lipids are vulnerable to oxidation which changes their structure and activities, it was suggested that GlcCer might undergo oxidation and alter its functions by modifying its structure. Generally, oxidation results in the loss of these beneficial properties. Conversely, an increase in activity has been reported in some cases (certain oxidants, such as oxidized phospholipids, prevent LPS-induced inflammation by inhibiting TLR4 activation^42^). These facts made us interested in whether the anti-inflammatory effects of GlcCer are altered by oxidation. In this study, as described above, we oxidized GlcCer in the presence of a photosensitizer and demonstrated that GlcCer was indeed oxidized by using qTOF-MS and LC–MS/MS. As a result, we obtained the mixture of GlcCerOOH isomers in which hydroperoxyl groups were attached to the double in the LCB of GlcCer (i.e., 8-OOH-GlcCer and 9-OOH-GlcCer). Then, we subsequently compared the anti-inflammatory activity of GlcCerOOH isomers with that of unoxidized GlcCer in differentiated Caco-2 cells treated with LPS to determine whether oxidation alters its actions.

**Figure 4:**
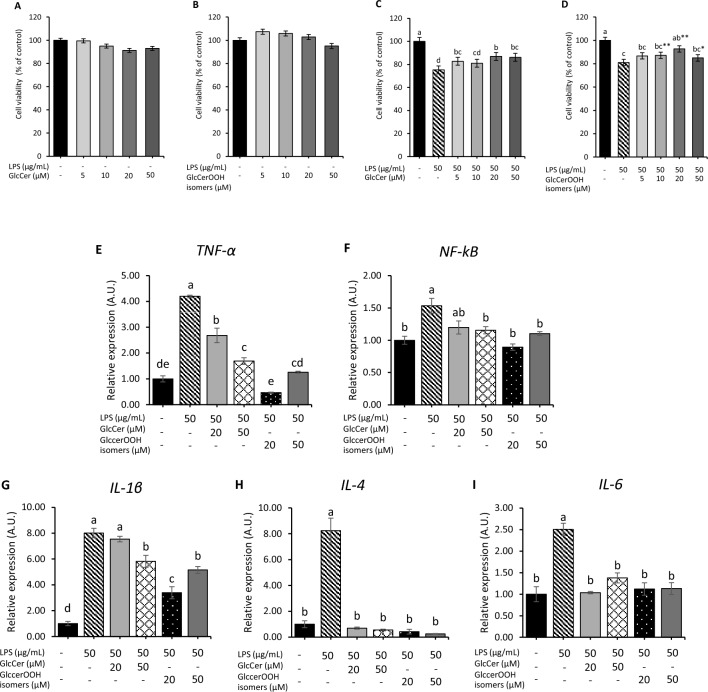
Effect of GlcCer and GlcCerOOH isomers on LPS-induced cell viability damage (**A**–**D**) and inflammation (**E**–**G**). Differentiated Caco-2 cells were treated with GlcCer or GlcCerOOH isomers in the presence (or absence) of LPS for 48 h, and cell viability was assessed. Similarly, cells were treated for 24 h, and inflammatory gene expression was measured. Details are shown in the Methods section. For cell viability, data are representative of mean ± SE, n = 12. Three independent experiments were performed in quadruplicate. For inflammatory genes, values are mean ± SE, n = 6. Three independent experiments were done in duplicate. Different letters (a–e) indicate significant differences between treatments (*p* < 0.05) compared to control, determined by one-way ANOVA with Tukey’s test. Note that differences between means with the same letter are not statistically significant. *Asterisks* on the bars (**D**) indicate significant differences from the corresponding groups (**C**) by *t-test* (*p* < 0.05).

**Figure 5:**
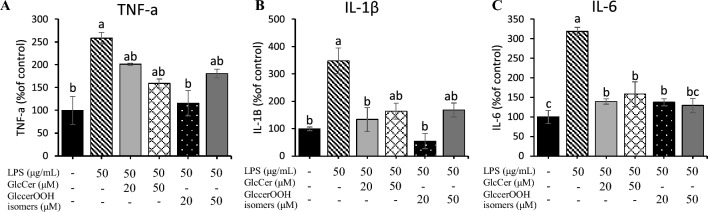
Effect of GlcCer and GlcCerOOH isomers on cytokine secretion on LPS-induced inflammatory stress. Differentiated Caco-2 cells were treated with GlcCer or GlcCerOOH isomers in the presence (or absence) of LPS for 24 h and cytokines secretion was measured. Details are shown in the Methods section. Values are representative of mean ± SE, n = 6. Three independent experiments were performed in duplicate. Different letters (a–c) indicate significant differences between treatments (*p* < 0.05) compared to control, determined by one-way ANOVA with Tukey’s test. Note that differences between means with the same letter are not statistically significant.

First, we confirmed that treatment of differentiated Caco-2 cells with 0–50 μM GlcCer for 48 h had no effect on cell viability (Fig. 4A). The GlcCerOOH isomers were also found to have no effect on viability (Fig. 4B). Since cytotoxicity has been reported at similar concentrations for other lipid hydroperoxides (e.g., linoleic acid hydroperoxide)^43^, it is possible that the cytotoxicity of GlcCerOOH is relatively low. Based on these results, we decided to perform the following experiments at concentrations of 0–50 µM GlcCer or GlcCerOOH isomers, where no cytotoxicity was observed. As a result, treatment of differentiated Caco-2 cells with LPS for 48 h significantly reduced cell viability (Fig. 4C, D). We found that not only GlcCer but also GlcCerOOH isomers significantly inhibited the LPS-induced cell injuries, in particularly at 20 μM treatment (Fig. 4C, D). These results clearly indicated that GlcCer does not lose its activity due to oxidation and highlight that oxidized GlcCer is not toxic to cells. Then, the expression of inflammatory genes (i.e., *TNF-α*, *NF-kβ*, *IL-1β*, *IL-4*, and *IL-6*), which are known to control the inflammatory response^44^, were evaluated by RT-qPCR. After LPS treatment for 48 h, a reduction in cell viability was detected. Thus, to identify the pathways of the phenomenon. In this study, we investigated gene expressions at 24 h after treatment. The RT-qPCR data revealed that LPS significantly increased the expression of *TNF-α*, *NF-kβ*, *IL-1β*, *IL-4*, and *IL-6* (Fig. 4E–I), and co-treatment of LPS with GlcCer or GlcCerOOH isomers was able to restore the expression of these genes to near-normal levels (Fig. 4E–I). GlcCerOOH at 20, 50 μM showed the suppression of the inflammatory cytokines in the relative gene expression levels. However, GlcCerOOH at 20 μM demonstrated the most effective suppression of inflammatory cytokines. Meanwhile, GlcCerOOH at 50 μM inhibited some inflammatory cytokines slightly but still established the potential to suppress the inflammation. Therefore, it is essential to highlight that the concentration of GlcCerOOH isomers plays a pivotal role in its impact on inflammatory responses. Furthermore, to confirm its anti-inflammatory activities, cytokine secretion levels in the cell culture supernatant were investigated. The results demonstrated that LPS treatment significantly increased TNF-α, IL-1β, and IL-6 compared to the control group. Meanwhile, co-treatment of LPS with GlcCer and GlcCerOOH isomers significantly decreased the cytokine levels (Fig. 5). These findings suggested that not only GlcCer but also GlcCerOOH isomers suppressed LPS-induced inflammatory stress via downregulation of inflammatory cytokines.

**Figure 6:**
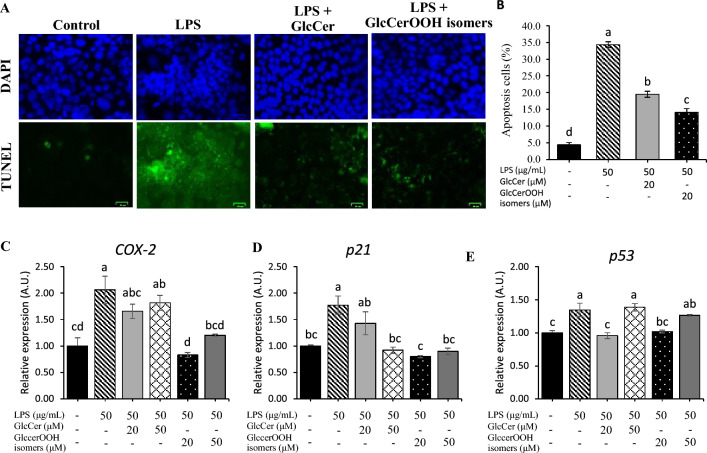
Effect of GlcCer and GlcCerOOH isomers on LPS-induced apoptosis. Differentiated Caco-2 cells were treated with GlcCer or GlcCerOOH isomers in the presence (or absence) of LPS for 24 h. Cell apoptosis was assessed under TUNEL and DAPI staining; data are representative of at least two independent experiments (**A**), the ratio of apoptotic cells was calculated (**B**), and apoptotic gene expression was examined (**C**–**F**). Details are shown in the Methods section. For apoptotic ratio data, values are mean ± SE, n = 9. Three independent experiments were performed in triplicate. For apoptotic genes, values expressed as mean ± SE, n = 6. Representative of three independent experiments performed in duplicate. Different letters (a–d) indicate significant differences between treatments (*p* < 0.05) compared to control, determined by one-way ANOVA with Tukey’s test. Note that differences between means with the same letter are not statistically significant.

To further understand the anti-inflammatory effects of GlcCer and GlcCerOOH isomers, apoptosis (e.g., formation of apoptotic bodies and typical chromatin) was evaluated by TUNEL assay and DAPI staining. Treatment of differentiated Caco-2 with LPS for 24 h induced apoptosis, as evidenced by a significant increase in the ratio of apoptotic DNA degradation and blebbing of the membrane compared to the control cells (Fig. 6A). As in the experiments on cell viability and inflammatory gene expression, co-treatment of LPS with 20 μM GlcCer or GlcCerOOH isomers significantly decreased the number of apoptotic cells (Fig. 6B). Additionally, apoptosis-related gene expression (i.e., *COX-2*, *p21,* and *p53*) were evaluated and results showed that GlcCer and GlcCerOOH isomers also suppressed LPS-induced *COX-2*, *p21*, and *p53* expression (Fig. 6C–E).

Generally, oxidized lipids are considered to be harmful and induce proinflammatory, whereas some lipids exhibit protective effects; cells and tissues respond to some oxidized lipids with activation of anti-inflammatory processes^17–19^. In this study, we investigated the direct effect of GlcCer and GlcCerOOH on intestinal epithelium and provided evidence that GlcCerOOH isomers suppress inflammatory gene expressions to exhibit protective properties in LPS-stimulated inflammation. An important anti-LPS mechanism is the mutually exclusive binding (antagonism) with TLR4 and its accessory proteins (e.g., MD-2, CD14, and LBP). Past studies indicated that lipid-A-like LPS^45^ and oxidized phospholipids^17–19,46^ could act as antagonists of TLR4 and suppress the subsequent occurrence of inflammatory stress. Gangliosides, which are glycosphingolipids, were reported to act as antagonists of TLR4^47^. Hence, GlcCer and GlcCerOOH isomers, which are one type of glycosphingolipids, may act as antagonists of TLR4 and suppress LPS-induced inflammation. Moreover, Bochkov et al.^17^ reported that the LPS inhibition was selective for oxidized phospholipids but not for unoxidized phospholipids. Also, aldehydes (i.e., 4-hydroxynonenal), which are one of the end lipid peroxidation products, could prevent activation of the NF-kB pathway and IL-1β by LPS^48^. Furthermore, our previous study revealed that higher polarity of sphingolipids showed a more potent anti-inflammatory effect^41^; the oxidation attaches a hydroperoxyl group into GlcCer to increase the polarity and may even enhance anti-inflammatory properties. Additionally, a previous study demonstrated that inflammatory stress stimulates sphingolipid metabolism and protects intestinal cells through the use of these metabolites^23^. GlcCerOOH, which has a strange structure for intestinal cells, may be strongly and quickly metabolized compared to GlcCer. Thus, it is thought that the anti-inflammatory mechanism of GlcCerOOH in intestinal epithelium is by action of GlcCerOOH itself and/or its metabolites.

Overall, these suggested the advantages of GlcCerOOH isomers to mitigate inflammatory responses, and the chemical structure modification of lipids, including GlcCer by oxidation, is possible to control their functions. However, actual intestinal inflammation is related to tight junctions, immune cells, and secretion of mucin and IgA, and GlcCer was reportedly associated with tight junctions and immune cells^49,50^. Therefore, more detailed in vitro and in vivo mechanism analysis will be necessary. Further experiments would be required to confirm this exact mechanism, including epidemiological studies in the future.

## Conclusions

Our study demonstrates the oxidation of GlcCer in the presence of a photosensitizer, producing the GlcCerOOH isomers as the major primary oxidation products. Specifically, GlcCerOOH isomers exhibited inhibitory effects on LPS-induced inflammation, suggesting their potential role as antagonists in impeding LPS recognition and thereby attenuating inflammation. This emphasizes the significance of structure–function relationships in oxidized lipids, particularly in modulating their biological activities. These findings are valuable guidance for utilizing GlcCer as a functional food component with potential implications in developing strategies to mitigate inflammatory responses. The elucidation of these mechanisms opens avenues for leveraging GlcCer to control inflammation, thus contributing to the advancement of therapeutic approaches in inflammatory conditions.

### Supplementary Information

Supplementary Information.

## Data Availability

Raw data were generated at Tohoku University. Derived data supporting the findings of this study are available from the corresponding author on request.

## Abbreviations

BSA: Bovine serum albumin
CCK-8: Cell counting kit-8
Cer: Ceramide
CID: Collision-induced dissociation
DAPI: 4′,6-Diamidino-2-phenylindole
DMEM: Dulbecco’s modified eagle medium
ESI: Electrospray ionization
FBS: Fetal bovine serum
FOX: Ferrous oxidation-xylenol orange
GalCer: Galactosylceramide
GAPDH: Glyceraldehyde-3-phosphate dehydrogenase
Glc: Glucose
GlcCer: Glucosylceramide
GlcCerOOH: Glucosylceramide hydroperoxide
HPLC: High performance liquid chromatography
IBD: Inflammatory bowel disease
IL-1β: Interleukin 1β
IL-6: Interleukin 6
LacCer: Lactosylceramide
LCB: Long-chain base
LC–MS/MS: Liquid chromatography-tandem mass spectrometry
LPS: Lipopolysaccharide
MRM: Multiple reaction monitoring
NEAA: Non-essential amino acid solution
NL: Neutral loss
p21: Cyclin-dependent kinase inhibitor
P/S: Penicillin–streptomycin solution
p53: Tumor protein p53
qTOF-MS: Quadrupole time-of-flight mass spectrometry
RB: Rose Bengal
RT-qPCR: Real-time quantitative PCR
TLR4: Toll-like receptor 4
TNF-α: Tumor necrosis factor α
TUNEL: Terminal deoxynucleotidyl transferase dUTP nick end labeling
