# Supplementary material for: Analysis of oxidized glucosylceramide and its effects on altering gene expressions of inflammation induced by LPS in intestinal tract cell models
Source: Sci Rep. 2023 Dec 18;13:22537. doi: 10.1038/s41598-023-49521-3 (PMC10728070; doi:10.1038/s41598-023-49521-3)
Supplement: Supplementary file 1 — Supplementary Information. [file 41598_2023_49521_MOESM1_ESM.pdf]

Supplementary Information

Analysis of oxidized glucosylceramide and its effects on altering gene expressions of inflammation induced by LPS in intestinal tract cell models

Mirinthorn Jutanom[1,2], Shunji Kato[1], Shinji Yamashita[3], Masako Toda[4], Mikio Kinoshita[3], Kiyotaka Nakagawa[*1]

[1] Food Function Analysis Laboratory, Graduate School of Agricultural Science, Tohoku University, Sendai, Miyagi 980-8572, Japan

[2] Department of Molecular Pathobiology, Faculty of Pharmaceutical Sciences, Kyushu University, 3-1-1 Maidashi Higashi-ku, Fukuoka, 812-8582, Japan

[3] Department of Life and Food Sciences, Obihiro University of Agriculture and Veterinary Medicine, Obihiro 080-8555, Japan

[4] Food and Biomolecular Science Laboratory, Graduate School of Agricultural Science, Tohoku University, Sendai, Miyagi 980-8572, Japan

[*]**Corresponding author:** Food Function Analysis Laboratory, Graduate School of Agricultural Science, Tohoku University, 468-1 Aramaki Aza Aoba, Aoba-ku, Sendai, 980–8572, Japan. Fax: +81-22-757-4417.

E-mail address: kiyotaka.nakagawa.c1@tohoku.ac.jp

Content of SI

Figure S1 Total ion current chromatogram of Q1 scan of GlcCerOOH isomer mixture.

Figure S2 Q1 mass spectra of unoxidized D-erythro-sphingosine and oxidized D-erythro-sphingosine.

Table S1 Analytical conditions used for Q1 analysis.

Table S2 Analytical conditions used for product ion analysis.

Table S3 Analytical conditions used for MS/MS MRM analysis.

Figure S1

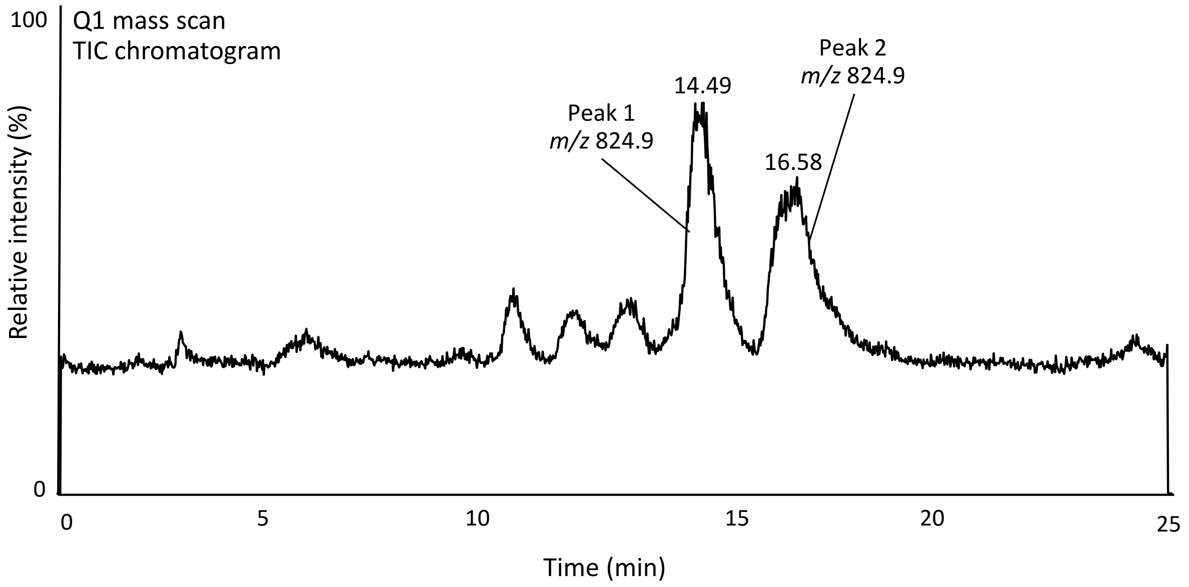

Figure S1 Total ion current chromatogram of Q1 scan of GlcCerOOH isomer mixture.

GlcCer was oxidized in the presence of RB at 4°C for 96 h under 18 W LED light of 50 Klux. The resulting sample was subjected to HPLC-UV (210 nm), and the GlcCerOOH isomer fraction was collected. The obtained GlcCerOOH isomer mixture was analyzed by LC-MS/MS. Details are shown in the Methods section. All peaks are represented by the mixture of GlcCerOOH isomers. For reference, peaks 1 and 2 are the GlcCerOOH (i.e., 8-OOH-GlcCer, 9-OOH-GlcCer) from the main component of rice-derived GlcCer (its purity is approximately 80%).

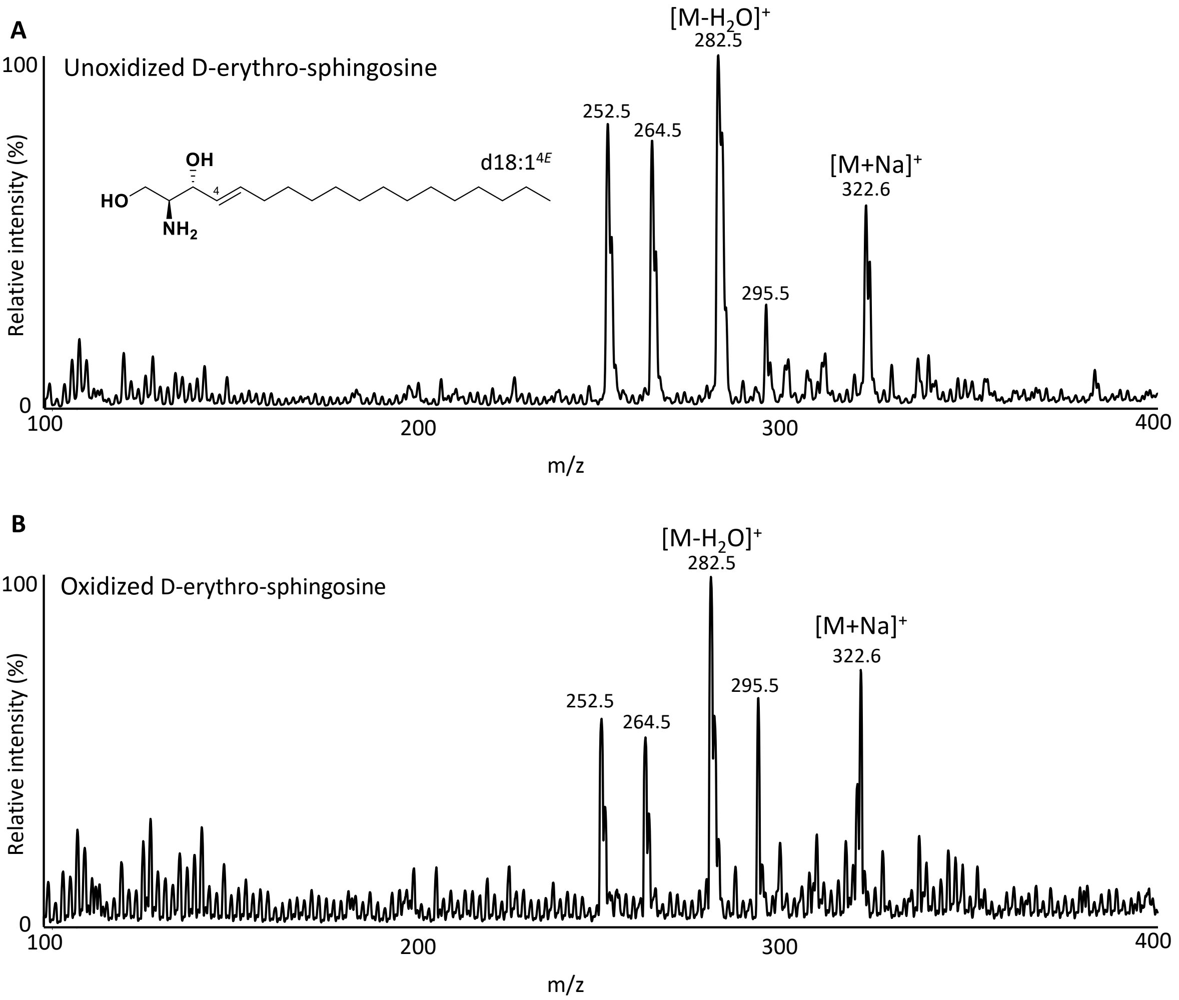

Figure S2 Q1 mass spectra of unoxidized D-erythro-sphingosine (A) and oxidized D-erythro-sphingosine (B).

D-erythro-sphingosine (d18:1^{4E}) (2 mg) was photooxidized under the same conditions as GlcCer. Before and after 24 h oxidation, the samples were infused into qTOF-MS to evaluate the oxidation of D-erythro-sphingosine. Details are shown in the Methods section. For reference, when oxidized D-erythro-sphingosine was measured by FOX assay, almost no hydroperoxide was detected. This result is considered to be in good agreement with the Q1 mass spectra results.

Supplementary tables

Table S2 Analytical conditions used for Q1 analysis

Parameters	GlcCer	Oxidized GlcCer
Source	ESI	ESI
Ion polarity	Positive	Positive
Mass range (*m/z*)	500-1000	500-1000
End plate offset (V)	500	500
Capillary (V)	4500	4500
Nebulizer (Bar)	0.4	1.6
Dry gas (L/min)	4	6
Dry temp (°C)	180	180
Funnel 1 RF (Vpp)	300	300
Funnel 2 RF (Vpp)	400	400
isCID energy (eV)	0	0
Hexapole RF (Vpp)	400	400
Ion energy (eV)	3	3
Low mass (m/z)	300	300
Collision energy (eV)	10	10
Collision RF (Vpp)	1000	1000
Transfer time (µs)	150	120
Pre pulse storage (µs)	10	10

RF, radio frequency

Table S3 Analytical conditions used for product ion analysis.

Parameters	GlcCerOOH isomers
Product ion (*m/z*)	824.8
Source	ESI
Ion polarity	Positive
DP (V)	125
EP (V)	10
CE (V)	50
CXP (V)	15
Curtain gas (psi)	20
Ion spray voltage (V)	5500
Temperature (°C)	600
Ion source gas 1 (psi)	40
Ion source gas2 (psi)	60
Collision Gas (psi)	4

DP, declustering potential; EP, entrance potential; CE, collision energy; CXP, Collision cell exit potential.

Table S4 Analytical conditions used for MS/MS MRM analysis.

Parameters	8-OOH-GlcCer	9-OOH-GlcCer
Precursor ion (*m/z*)	824.8	824.8
Product ion (*m/z*)	638.4	680.7
Source	ESI	ESI
Ion polarity	Positive	Positive
DP (V)	125	125
EP (V)	10	10
CE (V)	49	55
CXP (V)	17	14
Curtain gas (psi)	20	20
Ion spray voltage (V)	5500	5500
Temperature (°C)	600	600
Ion source gas 1 (psi)	40	40
Ion source gas2 (psi)	60	60
Collision Gas (psi)	4	4

DP, declustering potential; EP, entrance ptential; CE, collision energy; CXP, Collision cell exit potential.
